# An Automated Pipeline for Image Processing and Data Treatment to Track Activity Rhythms of *Paragorgia arborea* in Relation to Hydrographic Conditions

**DOI:** 10.3390/s20216281

**Published:** 2020-11-04

**Authors:** Ander Zuazo, Jordi Grinyó, Vanesa López-Vázquez, Erik Rodríguez, Corrado Costa, Luciano Ortenzi, Sascha Flögel, Javier Valencia, Simone Marini, Guosong Zhang, Henning Wehde, Jacopo Aguzzi

**Affiliations:** 1 Deusto Sistemas, 01015 Vitoria-Gasteiz, Spain; azuazo@deustosistemas.net; 2 Instituto de Cièncias del Mar (ICM-CSIC), E-08003 Barcelona, Spain; grinyo@icm.csic.es; 3 DS Labs, 01015 Vitoria-Gasteiz, Spain; vlopez@deustosistemas.net (V.L.-V.); erodriguez@deustosistemas.net (E.R.); javi.valencia.m@gmail.com (J.V.); 4 Consiglio per la ricerca in agricoltura e l’analisi dell’economia agraria (CREA)-Centro di ricerca Ingegneria e Trasformazioni agroalimentari, Via della Pascolare 16, Monterotondo, 00015 Rome, Italy; corrado.costa@crea.gov.it (C.C.); luciano.ortenzi@crea.gov.it (L.O.); 5 GEOMAR, Helmholtz Centre for Ocean Research Kiel, 24148 Kiel, Germany; sfloegel@geomar.de; 6 Institute of Marine Sciences, CNR, 19032 La Spezia, Italy; simone.marini@sp.ismar.cnr.it; 7 Stazione Zoologica Anton Dohrn, 80122 Naples, Italy; 8 Institute of Marine Research, N-5817 Bergen, Norway; guosong.zhang@hi.no (G.Z.); henningw@hi.no (H.W.)

**Keywords:** neural network, deep-sea, cold water coral (CWC), automated video imaging, filtering rhythms, tides, multivariate statistics

## Abstract

Imaging technologies are being deployed on cabled observatory networks worldwide. They allow for the monitoring of the biological activity of deep-sea organisms on temporal scales that were never attained before. In this paper, we customized Convolutional Neural Network image processing to track behavioral activities in an iconic conservation deep-sea species—the bubblegum coral *Paragorgia arborea*—in response to ambient oceanographic conditions at the Lofoten-Vesterålen observatory. Images and concomitant oceanographic data were taken hourly from February to June 2018. We considered coral activity in terms of bloated, semi-bloated and non-bloated surfaces, as proxy for polyp filtering, retraction and transient activity, respectively. A test accuracy of 90.47% was obtained. Chronobiology-oriented statistics and advanced Artificial Neural Network (ANN) multivariate regression modeling proved that a daily coral filtering rhythm occurs within one major dusk phase, being independent from tides. Polyp activity, in particular extrusion, increased from March to June, and was able to cope with an increase in chlorophyll concentration, indicating the existence of seasonality. Our study shows that it is possible to establish a model for the development of automated pipelines that are able to extract biological information from times series of images. These are helpful to obtain multidisciplinary information from cabled observatory infrastructures.

## 1. Introduction

Capturing deep ocean environmental features, habitat heterogeneity and associated biological components is increasingly required, especially in the light of growing impacts by human activities [1]. Despite the important role of the oceans within the earth system, marine ecosystems are significantly under-surveyed in time and space [2]. Our knowledge of species behavior, interspecific relationships and resulting community composition and biodiversity is incomplete in the deep-sea, due to difficulties in sampling [3]. High costs and logistic constrains in vessel-assisted surveys (e.g., by trawling and Remotely Operated Vehicle-ROV) prevent data acquisition at a high-frequency over continuous and prolonged time intervals [4].

Quantifying species behaviors and their variability at different spatiotemporal scales and how that variability affects the functioning of ecosystems and their productivity (e.g., the rate of transference of carbon matter and energy), requires the development of advanced customized hardware and software monitoring technologies [1,5,6]. One focal point for the establishment of those technologically advanced monitoring strategies is to increase our capability to track the organisms’ responses to changing oceanographic conditions in a remote, high-frequency and prolonged fashion [1].

Massive rhythmic movements by millions of individuals occur throughout the water column and on the seabed, as a result of the response of species to physical and biogeochemical cycles in light intensity and photophase duration as well as tidal currents. Those cycles are produced by the Earth–Moon reciprocal movement in relation to the Sun [7,8,9,10,11,12,13,14,15,16]. Many species have evolved time-keeping mechanisms (i.e., biological clocks) to cope and anticipate those predictable environmental changes [9,10]. For motile megafauna, crawling, walking and swimming rhythms can be characterized in terms of fluctuations in hauled or video-counted animals [6,10,16,17]. Differently, for sessile species such as Cold-Water Corals (CWCs), similar data for comparison are poorer. Hauling is potentially destructive, although some species survive the sampling procedure (increasing that survival chances with ROV operations). When colonies are transferred to laboratories, their activity can be tracked under the simulation of some environmental cycles such as currents, e.g., [18]. Alternatively, imaging should be adjusted in situ to portray polyps’ extrusion and retraction patterns, a technological solution being valuable to couple activity patterns to oceanographic control (e.g., hydrodynamism), substrate changes and time-related modifications of the faunal assemblages within CWC reefs [19,20,21].

Fortunately, imaging technologies are on the verge to be deployed on an increasing number of cabled observatory networks worldwide [4]. Artificial Intelligence (AI) routines such as machine learning can be used to survey the biological status of CWC from large image data sets [22]; for example, in situ high-frequency imaging from cabled observatory platforms such as the Lofoten-Vesterålen Observatory (LoVe; Norway) proved that the reef building CWC *Lophelia pertusa* displays activity rhythms over the 24-h range, where polyp extrusions are influenced by tidal currents [21]. Similarly, in the Stjernsund between Tromsö and Hammerfest, the gorgonian bubblegum coral *Paragorgia arborea* showed a similar tidal-oriented filtering activity [23].

To date, it is of strategic relevance to increment data collection capabilities of cabled observatories with autonomous image treatment and multiparametric data processing capabilities [6,24]. Monitoring autonomous functions needs to be developed through a pipeline of consecutive and automated steps for the acquisition, processing, validation and multivariate treatment of multiparametric data [12,25,26,27,28,29]. A major goal would be to pave the route to establish a real-time data processing and interpretation software toolkit, specifically designed for underwater observatories. That toolkit would enable the automated tracking of species behavior (and consequently rhythmic activity), providing the end-users of online statistic tools to evaluate the effects of different oceanographic variables (e.g., salinity, temperature, chlorophyll concentration, turbidity and water column depth as proxy for tides) to the biological response [6,24,27,30,31].

This study proposes an advanced AI-based Convolutional Neural Network (CNN) routine for the tracking of behavioral activity in the conservation-iconic *Paragorgia arborea* [32,33,34], to be used as baseline for the establishment of monitoring programs at the Lofoten-Vesterålen (LoVe) deep-sea observatory. Taking as inspiration previous computer science experiences and data treatments for this species in the area [21,23], we also acquired high-frequency time-lapse images of one coral colony with a fixed camera. We consequently established a CNN approach to evaluate colony changes in surface coverage in terms of percentages of extruded and retracted polyps as a proxy for the activity rhythm of the species. That approach was developed in a real-world context, where the marked oceanographic variability and operational monitoring challenges (e.g., light flickering and variable imaging angle) occurred [28]. Chronobiology-oriented statistics and advanced Artificial Neural Network (ANN) multivariate regression modeling were also used on all acquired behavioral and oceanographic data sets, to provide examples for further automation steps of multiparametric data treatment to be used to establish a pipeline of real-time treatment of ecological information at cabled observatories.

## 2. Materials and Methods

### 2.1. The Lofoten-Vesterålen (LoVe) Ocean Observatory

**Figure 1:**
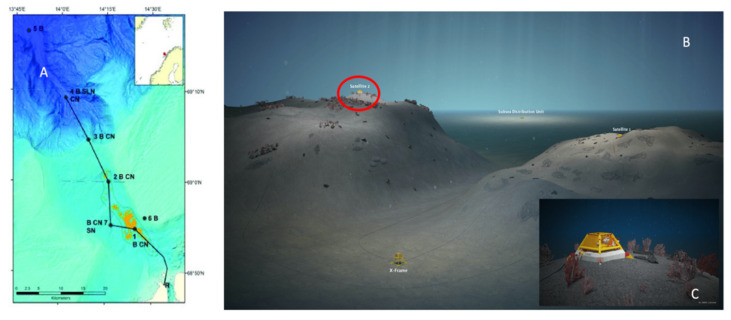
Overview of the Lofoten-Vesterålen (LoVe) canyon area (from Aguzzi et al. [6]). (**A**) The bathymetric map with the extended cabled transect with nodes (black dots) numbered 1–7 for shallower (light blue) to deeper realms (dark blue) and connected by a marine cable (continuous line). Node 1 is located in the shallowest part of the trough (orange areas represent the cold water coral (CWC) reefs). (**B**) A 3D detailed representation of the area around Node 1, where satellite no. 2 with camera appears on top of a small mound (encircled in red). (**C**) Enlarged view of the areas surrounding the satellite #2, where *Paragorgia* colonies are schematized.

The Norwegian continental shelf hosts some of the richest CWC reefs in the world [35]. A network of cabled video-observatories endowed with oceanographic multiparametric sensor technology (i.e., the Lofoten-Vesterålen, LoVe) was installed in the Hola Trough ~20 km off the north-west coast of the Lofoten Islands (Norway; Figure 1) [36]. LoVe has been in operation since 2013, to monitor the biological status of CWC mounds and their associated communities [37]. Node 1 (Figure 1A) is located in the south-eastern part of the Hola Trough at a depth of ~260 m, just south of the Vesterålsgrunnen bank, where CWCs are mostly constituted by *Lophelia pertusa* [38,39]. It has two satellite platforms and the #2 provided the imaging data set for this study (Figure 1B), being surrounded by *P. arborea* colonies (Figure 1C).

### 2.2. The Monitored Soft Coral Species

*Paragorgia arborea* (Linnaeus, 1758) is a semi-cosmopolitan deep-sea gorgonian [40,41,42,43,44], occurring on rocky and on dead coral frameworks, facing prevailing current [45,46,47]. This species occurs in fjords, continental margins, submarine canyons and seamounts [48,49,50]. Its arborescent morphology provides habitat and a niche to a wide variety of invertebrates [51,52] and fishes [53]. Due to slow growth and low recruitment rates, *P*. *arborea* is highly vulnerable to anthropogenic impacts such as trawling and long-line fishing [54,55] as well as sediment resuspension from bottom trawling or drill cuttings [56]. Future management plans should pay special attention to polyp behavior and its temporal expression in relation to the oceanographic factors, as an indicator of anthropogenic stress.

### 2.3. Image and Oceanographic Data Collection

The temporal activity of an individual colony of *P. arborea* was studied with 1-h time-lapse images acquired by a High Definition camera (Metas A.S.) from 23rd February (11:30) to 11th June (23:30) 2018. Although the camera was stereoscopic, only mono-images at a resolution of 2751 × 2206 pixels were used. A total of 6799 images were acquired. Two flashes on both sides of the camera illuminated the scene for 3 s prior to camera capture.

Oceanographic data such as temperature, salinity, turbidity, chlorophyll-*a* (hereafter named as chlorophyll) and water column depth (as a proxy for tidal bulges) were collected at 1 s frequency by sensors installed beside the camera (Table 1), in the same satellite #2 frame infrastructure (see Figure 1C). We selected the oceanographic data corresponding to the timing of each image. This simultaneous acquisition of oceanographic data is of relevance to enforce an experimental in-situ approach to the characterization of deep-sea species ecological niches [24]. In particular, one should notice that we used the following algorithm for the calculation of the chlorophyll concentration:

Chl a = 1.050 * Chl fl (day) + 1.0129 for daytime values

Chl a = 0.963 * Chl fl (night) + 0.2159 for nighttime values

where Chl and fl stand for chlorophyll and fluorescence. The LoVe observatory was under establishment at the time of the conducted measurements, so total values may be subject to change. In the present phase, the observatory focus is laid on the quality assurance of data.

### 2.4. Image Treatment

**Figure 2:**
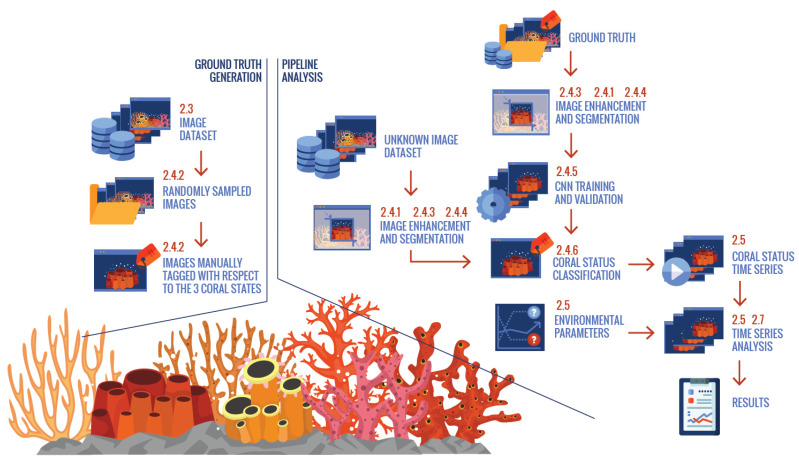
Pipeline for biological data extraction (via automated video-imaging procedures, based on Convolutional Neural Network (CNN) groundthruted routines) and analysis (via multivariate time-series treatment) of high relevance for advanced ecological monitoring functionalities by cabled observatories. Numbers on the upper right corner of each box (i.e., the treatment pipeline step) are referring to the numerals for the respective Materials and Methods section of this paper.

Different steps were implemented to extract the biological information on coral activity from images, based on extruded or retracted polyps (see below in Section 2.4.2). These steps were presented as a processing pipeline, to provide a model for the future implementation of automated procedures (Figure 2). All pipeline image treatment steps are described along with the terminal steps related to the time-series and multivariate statistical treatment and modeling.

#### 2.4.1. Image Enhancement

The images were usually very dark. Thus, different computer vision approaches were tested for enhancing the foreground/background contrast while simultaneously maintaining the original color, prior to the automated extraction of the morphological index (see next Section 2.4.2.). Several filtering procedures were evaluated as Gamma, Logarithmic, Sigmoid, as well as Histogram Equalization, Re-Scale intensity and Contrast Limited Adaptive Histogram Equalization (CLAHE). A total of 20 images were randomly selected to be filtered with all the mentioned methods. CLAHE was the best and only rendering technique that achieved both objectives.

#### 2.4.2. Supervised Tagging of Coral Areas

**Figure 3:**
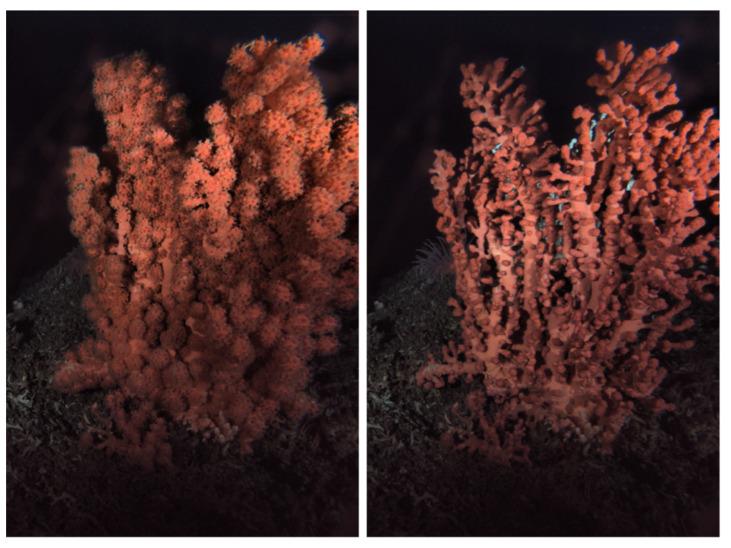
Different activity statuses for the coral as bloated (left; for polyp extrusion) and non-bloated (right; polyp retraction), as revealed after Contrast Limited Adaptive Histogram Equalization (CLAHE) image enhancement.

**Figure 4:**
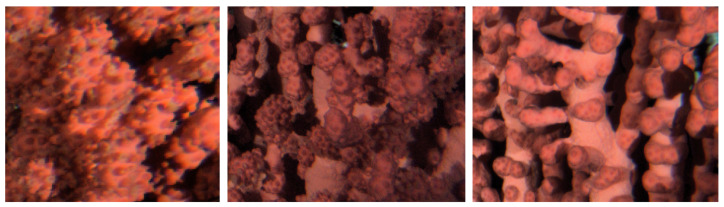
Different activity statuses of the coral as bloated (**left**), semi-bloated (**middle**) and non-bloated (**right**) for comparison. Those types of rectangular enlargements were selected in each image for the manual tagging of the behavioral status when establishing the training set.

Images of the coral were processed, in order to extract an index for polyp extrusion and retraction, as a proxy for colony activity. That activity pattern was indicated here as bloated at full polyp extrusion (e.g., likely during filter-feeding phases) and non-bloated, when all polyps were fully retracted with a transient semi-bloated phase of mixed colony behavior (i.e., with clusters of extruded and retracted polyps) [57,58,59,60,61].The two major statuses, bloated and non-bloated morphology, are presented in Figure 3 for the whole colony. A third transient semi-bloated status is also considered and shown in Figure 4 along with other bloated and non-bloated ones for comparison, within enlarged views.

A training dataset for the automated classification of the three coral states was created. That dataset was established by randomly choosing 1000 images out of the total (i.e., 6799 images). In each image, 3 different rectangular regions of the coral (such as those presented in Figure 4, as an example) were manually tagged, to determine the activity status, resulting in 3000 section images: 1376 bloated, 708 non-bloated, 916 semi-bloated. As the tagging was manually performed, the sizes of each of the rectangular regions was slightly different in size.

#### 2.4.3. Coral Segmentation

**Figure 5:**
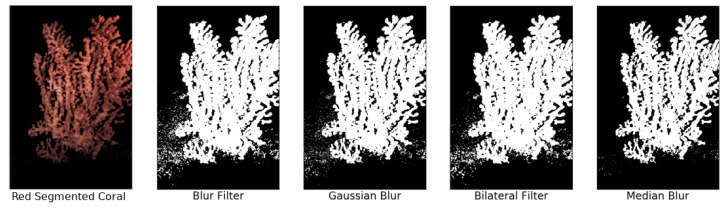
Red-segmented coral output and the image binarization according to the used filters.

A background/foreground image segmentation was implemented in order to improve the classification performance of the coral behavioral status in terms of area coverage by polyps in the extruded or retracted status. A color-based (red) mask segmentation by Hue Saturation Value (HSV) [62] was used for this approach. The images were transformed into HSV color space and the mask color ranges were set to extract the foreground coral region. Next, a bitwise comparison was made between the image and the color mask, producing a binary threshold image. The red-segmented color mask processing allowed for the extraction of the pixels belonging to the coral with a fast and low-intensive computational burden (Figure 5). Then, several filters were applied to empirically compare the segmentation results [63]: Blur, Gaussian Blur, Bilateral, and finally, Median. The different filter effectiveness is evidenced by image treatment outputs in Figure 5.

**Figure 6:**
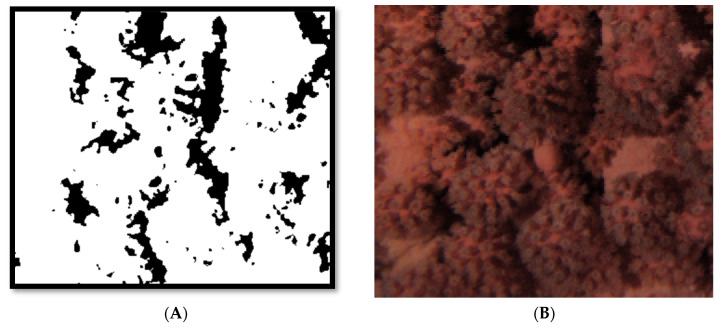
Output of coral section segmentation (**A**) and binarization with median filter (**B**).

The Median filter performed the better in terms of reducing white noise and preserving the details of the coral. A 3 × 3 kernel version of that filter was applied to the resulting images. For each of the 3000 rectangular section images, red mask coral segmentation was applied (Figure 6).

#### 2.4.4. Data Preparation

Coral segmentation with a color mask allowed for the pre-processing of each of the manually tagged sections of the coral. For each of these regions, the segmentation techniques were applied (see above). From each segmented image, 120 pixels belonging to the coral (white pixels) and 40 pixels belonging to the background (black pixels) were randomly selected and encoded into a text file together with a pixel label: bloated, semi-bloated, non-bloated and background. Thus, for each image, 160 pixels were stored.

#### 2.4.5. The Convolutional Neural Network (CNN) Model

To characterize the temporal expression of polyp behavioral rhythm (i.e., bloated, semi-bloated and non-bloated), a CNN model [64] was implemented to segment and hence identify the pixels belonging to each behavioral category, as follows. A six-layer CNN was trained with image pixel annotations and the corresponding images from the previous training stage. For each of the annotation pixels, a 30 × 30 crop image was created from its corresponding coral image where the middle pixel was the annotation pixel, following a strategy similar to Patches-GT [65]. This strategy is based on expanding the labeled ground truth pixels into labeled patches around those pixels. It assumes that the surrounding pixels of a labeled one are identical. Each of these cropped images was labelled with its corresponding pixel class. These cropped images and their labels were used as training/test data.

**Figure 7:**
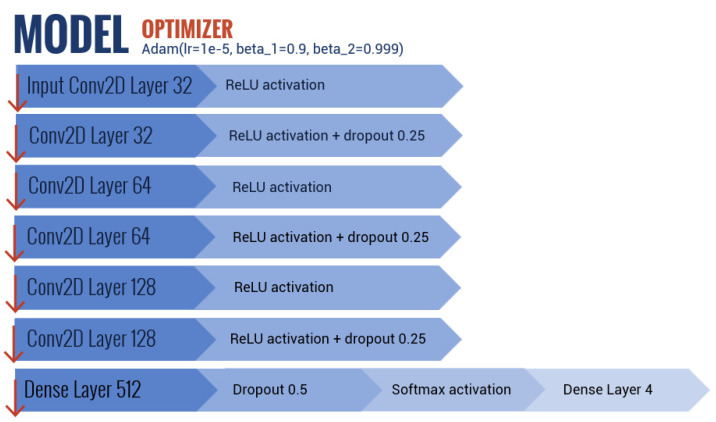
Structure of the proposed CNN.

The CNN was implemented with a Keras library [66]. The proposed CNN (Figure 7) was composed of: a convolutional layer with 32 outputs and an input shape of (30,30,3); another convolutional layer with 32 outputs; a dropout layer with a rate of 0.25; a convolutional layer with 64 outputs; another convolutional layer with 64 outputs; a dropout layer with a rate of 0.25; a convolutional layer with 128 outputs; another convolutional layer with 128 outputs; a dropout layer with a rate of 0.25; a dense layer with 512 outputs; and finally a final dropout layer with a rate of 0.5 plus a dense layer with 4 outputs. The last dense layer had a value of 4, being equal to the number of statuses to classify (i.e., bloated, semi-bloated and non-bloated) plus the background. All the convolutional layers had 3 × 3 filters and the Rectified Linear Unit (ReLU) activation functions, except for the last dense layer, which had a Softmax activation function. Even convolutional layers had MaxPooling2d with a pool size of (2,2).

As for the optimization used to train the model, several variations of the algorithm ADAM were compared through trial and error. The best algorithm used a learning rate of 0.0001, beta_1 of 0.9 and beta_2 of 0.999. These parameters allowed the CNN to converge faster. The model was compiled with a loss function of “categorical_crossentropy” [67], since a non-binary class problem was addressed.

Data augmentation was used on the train/test crop images to have a much wider dataset that would yield a larger representation of coral shapes, by horizontal and vertical flips as well as width and height shift ranges of 0.1. A 10-fold cross-validation [68] was used to test the model. Each fold was stratified and randomized, in order to preserve the percentage of samples of each class. Within the 10-fold cross-validation, 9 folds were used for training and 1-fold for validation, repeating the experiment 10 times, by changing the validation fold each time (and as consequence the training folds). The final performance was the average of all the single validation performances. A batch size (number of training samples utilized in each iteration) of 256 was set for the training. The network was trained with 100 epochs (number of times the algorithm works through the training dataset).

#### 2.4.6. The Segmentation of Coral Behavioral Statuses by CNN Modelling

The procedure to segment the images was performed via 30 × 30 window crops and yielded to the classification of coral behavior into the 3 categories plus the background (see Section 2.4.2. above). As we needed to iterate the pixel matrix of high-resolution images, the required computational time would have been too high (3000 s in average). In order to create a lighter computational tool, columns and rows were iterated with a step-width of 10. With this approach, the processing time of each image was reduced to ~75 s. This approach allowed us to create a map over the image in which each classified 30 × 30 window could partially overlapped other windows [69]. This procedure created a more realistic and precise map to interpret the activity status of the coral.

### 2.5. Behavioral and Environmental Time Series Compilation

The CNN automated approach enabled us to quantify the coral activity status from all considered images. Once the segmentation of each image was concluded, we could classify and count the pixels belonging to bloated, semi-bloated and non-bloated categories. In order to calculate an overall percentage of activity statuses for the whole coral colony, the pixels belonging to the three behavioral categories were summed up. Then, for each status, the sum of its pixels was divided between the total amount of coral pixels considered, obtaining a relative percentage.

Finally, a final dataset was created based on environmental and oceanographic data (as collected at times corresponding to images) as follows: date, time of the day, overall percentage of colony in bloated, semi-bloated and non-bloated status, readings for temperature, salinity, turbidity, chlorophyll, and depth.

### 2.6. Time Series Analysis

The occurrence of significant periodicities in all temporal data sets was analyzed with the Lomb–Scargle periodogram by the El Temps chronobiology software package (www.el-temps.com). All periodicities were screened in a window of frequencies between 600 and 1620 min (equivalent to 10–27 h, respectively), encompassing ranges for diurnal and semi-diurnal tides plus the day–night cycle [16]. In the periodogram output plot, the peak exceeding a significant threshold (*p* < 0.05) represented the maximum percentage of total data variance, explained by the inherent dominant periodicity [70].

Waveform analysis for the 3 coral activity statuses and the oceanographic data was performed to identify the phase (i.e., peaks timing) of rhythms and cycles [13]. All time series were subdivided into 24-h segments with a 1-h sampling frequency. All values were averaged at corresponding 1-h time intervals throughout all segments. Resulting averages (plus standard deviations) were displayed in 24-h plots (i.e., the waveform), along with a Midline Estimated Statistic of a Rhythm (MESOR). This is a daily average, obtained by re-averaging all waveform values [70]. In the waveform plot, the phase of a rhythm (i.e., the averaged peak) is identified by all values above the MESOR. Waveform plots for the three coral activity statuses were represented with intermediate dusk and dawn timings at the 23rd of April 2018 (i.e., considering the 5-months’ extension of the image acquisition period), as indicated by the local astronomical web service (https://www.timeanddate.com/sun/norway/bergen): dusk, 21:30 and dawn, 6:00. This plot implementation was done in order to show the linkage of detected rhythms upon the light intensity cycle.

### 2.7. Multivariate Modeling of Coral Activity

An AI classification approach was used to predict the activity status of the coral, considering a global binary behavior for the colony, with 2 statuses as bloated and non-bloated (semi-bloated was merely transient) [21,71]. We considered 7 independent and conditioning variables (i.e., the input x-block): time of the day (reported as a unit fraction of 24 h), chlorophyll, temperature, depth, turbidity, salinity and the Field Of View (FOV). The latter is a binary dummy variable, while the remaining 6 are continuous. The continuous variables were standardized (i.e., centered with respect to their mean and scaled by the relative standard deviation). The dependent response variable (y-block) was the bloated percentage of each image, discretized into a binary variable (i.e., bloated, not-bloated); when the percentage of the bloated surface was higher than or equal to 50%, it was converted to 1, and, when lower, it was converted to 0.

The x-block was trained several times using shallow neural networks for binary classification with different hidden layer sizes. If no convergence was reached due to underfitting, the number of features was increased by considering the square and cubic terms of the x-block obtained by multiplying its columns in a combinatorial fashion. In order to mitigate the overfitting, the cross-validation Root Mean Square (RMS) error was calculated as a function of the number of features. Thus, 133 features were obtained and used to train a Multi-Layer Feed forward artificial neural Network (MLFN; [72,73]) with a single hidden layer architecture, using sigmoid hidden and SoftMax output neurons. The ANN-based MLFN approach was trained with the Bayesian regularization back propagation algorithm [74,75], as implemented in the deep learning MATLAB toolbox. The dataset was partitioned using 70% of the observations (N = 1150) as training set and the rest as test set (N = 503). The test set was used to validate the model. This partitioning was optimally chosen with the Euclidean distances, selecting parameters without a priori knowledge of a regression model. The cost function was minimized using the normalized RMS error performance function with a 10^−10^ threshold on the gradient. Precision (P) and Recall (R) were also calculated on training and test sets.

In order to extract the most informative features, among the 133 acquired ones, a feature importance analysis was conducted as follows [76]. The hidden layer matrix (9 nodes x 133 variables) was a posteriori analyzed by considering its elementwise absolute value. From this matrix, the maximum value for each variable (i.e., column) was extracted, obtaining a 1 × 133 row vector. The top 15 most significant variables were chosen. The larger the value, the more relevant the contribution to the MLFN model. The model was developed by using the MATLAB 9.7 R2019b Deep Learning Toolbox.

Having the phenomenon of an activation shape, a comparison between the real percentage of bloated coral surfaces and the sigmoid of the most relevant standardized quantitative variables (i.e., time of the day, chlorophyll, salinity, temperature, depth and turbidity) was made. Pearson linear correlation and their probability were estimated.

## 3. Results

### 3.1. CNN Coral Segmentation: Evaluation of Training and Performance

**Figure 8:**
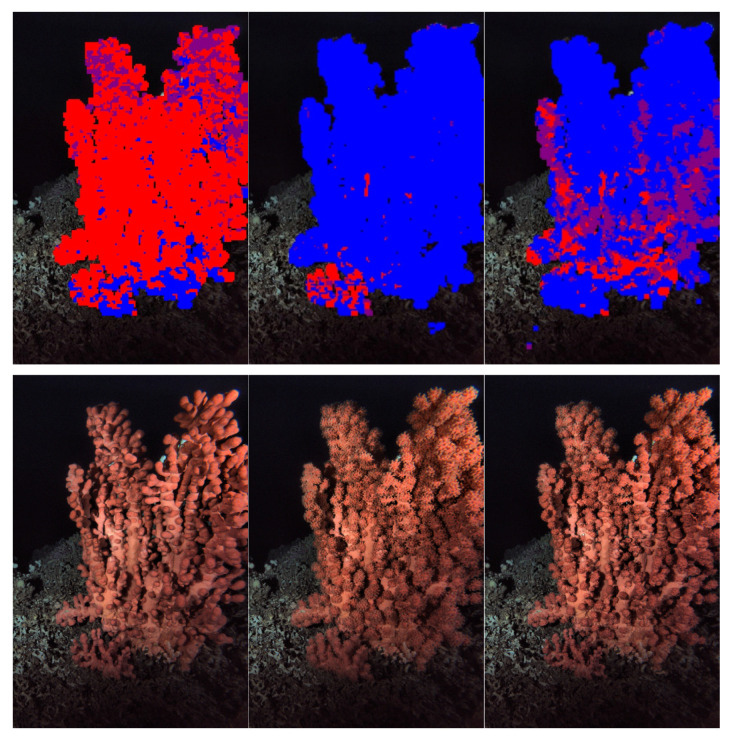
Examples of coral segmentation indicting activity status (**above**) and original images (**below**). Non-bloated areas are in red and bloated ones are in blue. Purple areas are in the transient semi-bloated condition.

After the CNN was trained, 3000 images were segmented via a 30 × 30 crop window classification, in order to have at least one value of coral activity status per one hour. Bloated pixels of the coral were visualized as the blue color, semi-bloated pixels as purple and non-bloated ones as red (Figure 8). The resulting CNN was used to segment the remaining images from the time series. The CNN model had an average test accuracy of 86.16% and an average Area Under Curve (AUC) score of 0.9732 in the 10-fold cross-validation of the model.

After the 10-fold cross-validation, the CNN model was trained with a 70/30 split in the dataset. This meant that a random 70% was used as training data, while the 30% left was used as test data. The CNN gave a final test accuracy of 78.08% and a validation AUC of 93.47%. The obtained Precision Accuracy (PA) and User Accuracy (UA) are shown in Table 2.

### 3.2. Time Series Analysis

**Figure 9:**
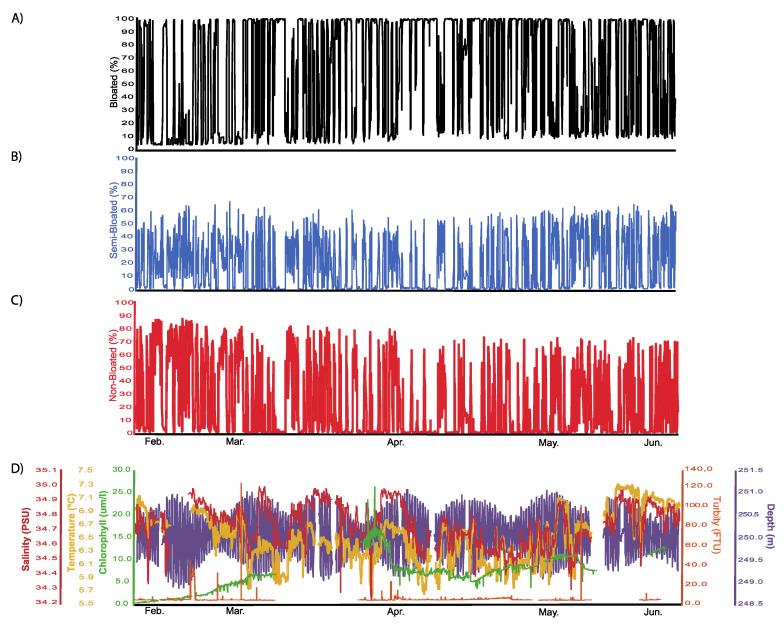
Time series of automated percentages of colony surface with polyps in full extrusion (bloated; (**A**)) or full retraction (non-bloated; (**C**)) status as well as the intermediate condition with patches of both (semi-bloated; (**B**)), plus the concomitantly monitored oceanographic variables for comparison (**D**).

Time series analysis was performed on coral activity data (percentage of colony surface coverage by extruded and retracted polyps) as a final step in the pipeline of information treatment (see Figure 2). During the study period, polyp activity was registered continuously in terms of the percentage of colony surface, showing a bloated, non-bloated and semi-bloated status (Figure 9). Colony surface coverage values for bloated and non-bloated statuses attained maximum values close to 100% and 90%, respectively (Table 3; Figure 9A,C). Differently, the semi-bloated condition attained lower maximum area coverage values, close to 70% (Figure 9B). The oceanographic data contained large gaps as a product of transient sensor malfunction and pauses in measurement activity during platform maintenance (Figure 9D).

Periodogram analysis outputs for time series of those percentages of colony coverage in bloated, non-bloated and semi-bloated statuses are presented in Table 3. Significant 24-h patterns were reported for all three activity variables. No analysis was conducted on time series of oceanographic data, since those data were non-continuous.

**Figure 10:**
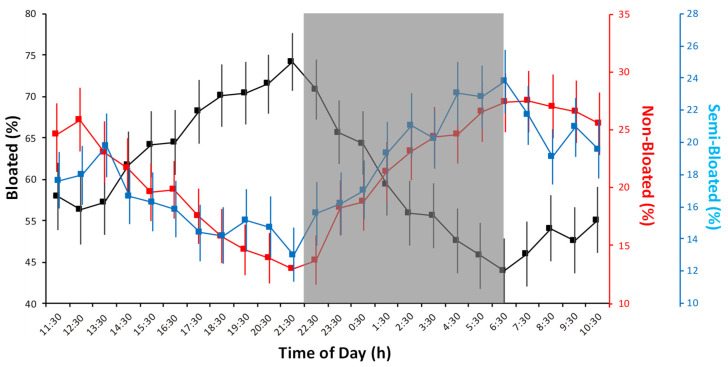
Waveform analysis outputs as averaged percentages for colony surface with polyps in full extrusion (bloated) or full retraction (non-bloated) as well as the intermediate condition (semi-bloated) are reported with the standard deviation. A marked unimodal increase in bloated status (i.e., possibly corresponding to filter feeding activity) is evident in antiphase with non-bloated and semi-bloated statuses (see the MESOR threshold values in Table 3). On should notice that waveforms *Y*-axes have different scales to make time series pattern as visually comparable. The gray shaded area represents the hours of darkness.

Waveform analysis of the three different coral activity statuses are presented in superposition to show their phases relationship (Figure 10). One should notice that the temporal pattern depicted by this graphic output sustains periodogram results (i.e., a single large peak per 24-h cycle; see Table 3). That combined and overlapped plotting evidenced the occurrence of a bloated peak towards dusk (and, consequently, a drop in non-bloated percentages at that time). Waveform values above the MESOR, showed for the bloated time series a significant increase from 14:30 to 00:30 (i.e., the peaks duration corresponds to all values above the MESOR). Conversely, the non-bloated status peaked from 2:30 to 14:30. Similarly, the semi-bloated condition showed a significant increase from 1:30 to 10:30 (with an isolated significant increase also at 13:30).

**Figure 11:**
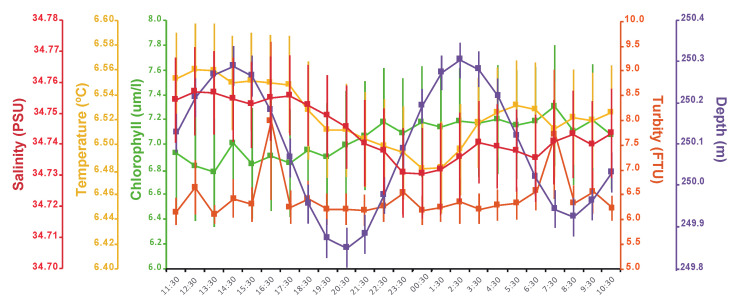
Waveform analysis outputs of oceanographic data, where all hourly averaged values are presented with their standard deviation.

The same waveform analysis was also performed for the oceanographic variables (Figure 11), to show the of phases relationships among their cycles. Considering MESOR values (not plotted but reported in Table 3), the marked bimodal water column depth variations (as proxy for tidal current regimes) are evident (two peaks: 11:30–15:30 and 0:30–5:30). This hydrodynamic pattern affected turbidity, which also showed two major sharp increases over 24 h, slightly delayed in comparison to water column peaks (16:30 and 07:30). Conversely, chlorophyll showed a high variability (wide SD bar ranges) over the 24-h cycle. Salinity and temperature were also very variable over the 24 h and peaked as follows: salinity between 11:30 and 20:30 and temperature between 10:30 and 18:30.

**Figure 12:**
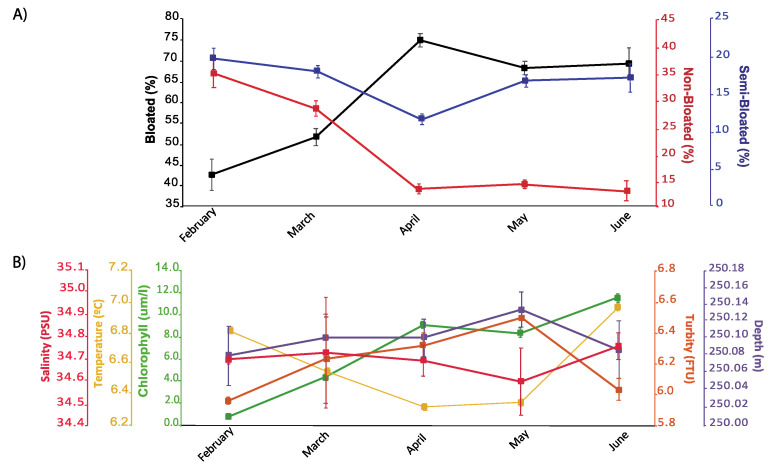
Seasonal pattern of variation in *P. arborea* polyps’ activity and concomitantly measured oceanographic variables. (**A**) Monthly average percentages of different polyp activity statuses. (**B**) Monthly averaged oceanographic variables. All data are presented with the standard deviation.

Some clues about the occurrence of seasonal trends in polyp extrusion activity were found during our monitoring period (Figure 12). This trend was evidenced by the computing of monthly averages in polyps’ extrusion activity in comparison with the oceanographic variables. A significant increase (considering MESORs values in Table 3) in overall activity occurred in April, May and June, matching a similar pattern for chlorophyll and turbidity. Monthly values of chlorophyll for April, May and June were significantly higher than those registered in February and March (PERMANOVA, PSEUDOF: 474, *p* = 0.001). For monthly turbidity values, a significant increase was found from April to May (PERMANOVA, PSEUDOF: 3177, *p* = 0.02).

### 3.3. Multivariate Modeling of Coral Activity

**Figure 13:**
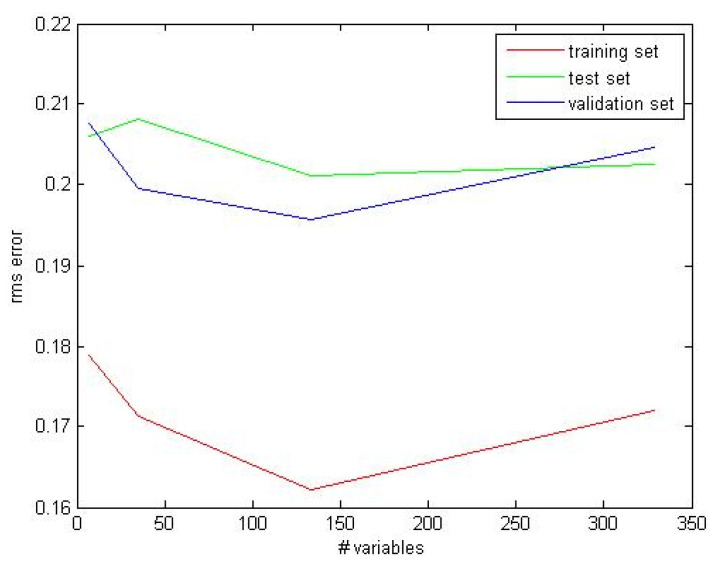
Root Mean Square (RMS) error, as a function of the number of variables for training, test and validation sets. The results were obtained averaging over 10 random trials.

Multivariate statistic treatment and modelling were performed as a final step for our automated data treatment pipeline (see Figure 2), to evaluate the response of biological data with respect to ambient environmental conditions. Our choice for the ANN class modelling was based on its feasibility for automation when the phenomenon to be analyzed has a non-linear temporal dynamic. In Figure 13, the RMS error, as a function of the number of features, is reported for training, test and validation sets. Since the cross-validation curve has a minimum at 133 features, we have chosen this value to perform the following calculations. The results were obtained averaging over 10 random trials.

Only 1653 observations (from 2nd February to 8th June 2018) were processed out of 3000. The remaining behavioral data were eliminated because they occurred during a gap in oceanographic data acquisition (see Figure 9D). The algorithm converged after 500 iterations with 0.26% of false predictions during training of the network and 31% of wrong predictions during tests. The output of the “softmax” function is a real number 0 < *p* ≤ 1, which represents the modelled probability of the coral to be bloated. Table 4 reports the principal results of the MLFN model used to predict bloated vs. non-bloated corals on the base of 133 variables (x-block). The confusion matrixes of the training and test sets are reported in Table 5. In the training set, only three out of 1150 (0.26%) were wrongly classified. During testing, 156 out of 503 (31%) samples were misclassified. Among these 156 + 3 (159) misclassified observations, 30 (18.9%) showed a percentage of bloated coral surface ranging from 20% to 80%. In this large interval (20–80%), there were only 197 observations (11.9%) of which 30 were misclassified (15.2%). In the remaining intervals (0–20% and 80–100%) the error was much lower (8.9%). This confirmed the dominant binary character of coral behavior, where polyps are globally open (bloated) or retracted within the calyx (non-bloated) in an almost synchronous mode. An intermediate state (semi-bloated) is only reached for a very limited time, thus confirming the appropriateness of the classification approach. The precision (P) values of the training and test sets were, respectively: 0.999 and 0.721. The recall (R) values of the training and test sets were, respectively: 0.997 and 0.761.

Considering the feature importance analysis, conducted to understand which variable or combination of variables had a higher impact on the constructed MLFN model for coral activity response, the top 10 features were the following: temperature^2^, salinity^2^, chlorophyll × salinity, hour × depth/pressure × FOV, hour × salinity, hour × temperature x salinity, depth × salinity, chlorophyll^2^ × depth, depth^3^, depth × salinity × FOV.

**Figure 14:**
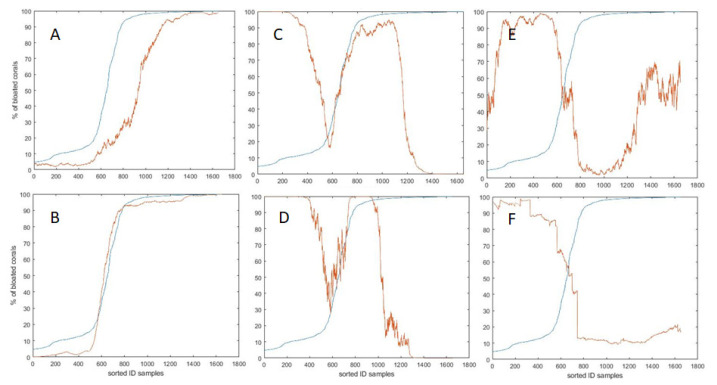
Samples sorted by increasing percentage of bloated coral status (saturation blue lines curves). The sigmoidal function of the centered and standardized oceanographic variables (red lines) are: (**A**) time of the day, (**B**) chlorophyll, (**C**) salinity, (**D**) temperature, (**E**) depth, and (**F**) turbidity. Before processing the oceanographic standardized variables with the sigmoidal function, a moving average was applied in order to smooth their signal.

Due to the structure of the x-block, the contribution of the continuous features (time of day, chlorophyll, temperature, depth, turbidity and salinity) was difficult to assess. On the other hand, given the binary attitude of coral behavior, samples were arranged for plotting in a saturation curve with a sigmoidal shape, to show an activation profile, i.e., the progressive percentage of bloated coral surface *versus* the growing status of the environmental variable. Figure 14 shows the comparison between the real percentage of bloated coral surface and the sigmoid of each standardized quantitative variable. One should notice that, if sorted accordingly, the standardized variables’ time of the day and chlorophyll showed an increasing linear relationship. As can be seen, the sigmoid of the time of the day (A) and chlorophyll (B) significantly correlate with the bloated status of the coral, *r* = 0.87 (*p* < 0.001) and *r* = 0.99 (*p* < 0.001), respectively. On the other hand, the sigmoid of the depth (E) and turbidity (F) are significantly anti-correlated with the bloated status of the coral; *r* = −0.78 (*p* < 0.001) and *r* = −0.99 (*p* < 0.001), respectively. The sigmoid of the temperature (D) is significantly correlated with the bloated status of the coral derivative: *r* = 0.17 (*p* < 0.001). The sigmoid of the salinity (C) is not correlated.

## 4. Discussion

In this study, we proposed a new pipeline for the extraction of biological information referring to the activity status of a sessile organism, the iconic bubblegum deep-sea coral species *P. arborea*, based on automated segmentation and CNN processing. That pipeline was also implemented in two relevant steps for time series statistic treatment for cabled observatories multidisciplinary data, based on chronobiology and multivariate statistics. This study indicates a new route for the further development of online tools for real-time processing of complexly interrelated multiparametric bio- and oceanographic data sets, which is still missing in most of the data management structures of cabled observatories today. Such an effort is at the core of our future capability to efficiently establish monitoring and surveillance programs with those marine infrastructural assets [24,26].

With the proposed pipeline for information extraction and data processing, we evidenced a tidal-independent and crepuscular activity pattern. A previous work on *P. arborea* image treatment showed tidal rhythms in polyp activity through the manual analysis of images for activity status classification [23]. Moreover, previous authors used logistic regressions (combined with the Recursive Feature Elimination), to decide which oceanographic variables were significantly linked with coral activity. Here, we used chronobiology-oriented statistical approaches (i.e., periodogram and waveform analyses), combined with an advanced Artificial Neural Network (ANN) modelling statistic. With this data treatment pipeline, we showed that the diurnal coral extrusion rhythm is phased at dusk and it is therefore strongly influenced by the time of the day rather than the tide status. Apparently, the difference in results between both studies can be attributed to increased analytic precision obtained with automated CNN routines in comparison to the manual processing of images.

### 4.1. Implications of a CNN-Based Pipeline for Ecological Information Treatment at Cabled Observatories

Cabled observatory cameras are still used for temporally scattered studies with no ambition for establishing institutionalized monitoring programs. This is because very few imaging devices are capable of hosting intelligence on board for extracting time series of biological data in a fully autonomous fashion [27,30]. This study indicates a new way of using optical data to link biological processes to the environmental forcing, by using a CNN-based automation procedure in the extraction of biological information from temporally extensive sets of acquired images. The tailored CNN procedure was a simple segmentation tool designed for this local experiment based on a 6-layer classification CNN. For the moment, no comparison studies were made with other segmentation approaches, such as CNN-based or Fully Convolutional based Network (FCN-based) approaches. However, CNN efficiency was tested, showing to be reliable for the objectives described in [77]. The 78.08% test accuracy and 93.47% AUC value are promising results even though user precision and product precision results could be improved with more balance between all classes.

Future studies should include a comparison with state-of-art approaches, such as Mask R-CNN [78] and other FCN architectures [79]. Furthermore, if more public datasets of deep-sea *Paragorgia arborea* become available, one should try using different colonies in different locations to achieve a more complete and adaptable segmentation tool.

Here, we efficiently used that image filtering and reconstruction procedures prior to CNN treatment. The artificial background illumination within the FOV varied in its intensity over consecutive photos for the flickering of the lighting system and also for the absorption and scattering effects caused by suspended particulate (i.e., turbidity). Many approaches were proposed in the scientific literature for improving the quality of underwater images, e.g., [80,81,82,83]. Restoration methodologies are based on the physical model of the light propagation and are used for correction of the effects of light absorption and scattering [84,85,86]. Contrastingly, enhancement methodologies do not assume any physical model and are based only on computer vision approaches for improving the light and color distribution and for reducing the hazing effects due to suspended particulate load [87,88,89,90].

The variation of the underwater acquisition conditions (e.g., light distribution and water turbidity) and the huge variety of marine fauna with respect to shape and size are the most relevant factors affecting the performance of the automated recognition and classification of underwater visual data [91,92]. All this differentiated information can be successfully managed by the supervised machine learning approach, where a set of examples representing all the information needed for discriminating the relevant specimens, can be used for training of the automated algorithms for recognition and classification [93,94]. The relevant information in visual data (i.e., polyp extrusion and retraction statuses as proxy for activity rhythms) was recognized and classified by combining computer vision and artificial intelligence methodologies after supervised training [95]. That training was efficiently carried out on a set of manually classified quadrants with only a reduced number of images, resulting in a performance that is comparable to that of a visual inspection operated by expert users.

The lack of automation in image treatment is strongly constraining the extraction of information from marine imaging devices [4,92] and, unfortunately, a universal customization is not feasible [27,30]. This situation is impairing the use of remote monitoring by cabled observatories to track changes in the status of ecosystems at high ecological levels of complexity (i.e., animals and the services they provide) [12]. Fortunately, the scientific literature is becoming rich in consolidated and emerging methodologies on computer vision and pattern recognition, e.g., [96,97,98,99]. CNN methodologies are becoming diverse, offering several customized examples of innovation for specific applications and environmental contexts (reviewed in [28]). A more innovative step forward is the evaluation of CNN procedures within the context of entirely automated pipelines for data treatment at cabled observatories, as the one we have drawn here (see Figure 2; [29]). Those pipelines should be considered and debated in relation to the feasibility to create future cyber-infrastructures including advanced digital libraries for storing, accessing and processing the data [24].

### 4.2. Functional Explanation of the Detected P. arborea Polyps’ Extrusion and Retraction Rhythms

Here, we report *P. arborea* polyps’ extrusion and retraction rhythms associated to the day-night rather than the tidal cycle, with activity peaking form the second half of the day toward a maximum at dusk. Anthozoan polyp extrusion has been positively correlated with current velocity [100], food availability or water temperature [101]. In deep-sea aphotic realms, activity rhythms relationship with current regimes are due to organic carbon transport [102]. In the LoVe deployment area, strong tidal cycles were supposed to drive the polyp extrusion of the CWC *Lophelia pertusa* [21]. It has been known for decades that the Lofoten area is a region of high mesoscale activity and associated to the amplification of eddy kinetic energy currents, i.e., [103]. The tidal forces mainly impacting the area are the semidiurnal lunar and solar tides and, although a factor smaller, by the diurnal lunar and solar ones (i.e., [102]). Despite the high variability in the circulation pattern within the area, the impact of hydrodynamics on the short-term behavior of the bubblegum coral seems to be limited, and, because of that, no deeper analysis was conducted on time series of oceanographic data.

Our results suggest a synchronization of *Paragorgia* polyps’ activity upon solar background illumination rather than tides. High-energy monochromatic blue radiation is still present even at depths greater than 250 m (depending on local turbidity conditions [103]). The benthic community at the LoVe deployment area may therefore be influenced by solar light-driven intensity variations. Our reported polyps’ activity could be related to the 24-h intermittent presence and absence of predators (e.g., pelagic amphipods and euphausiids; [104]) that could be visually lurking on the coral surface at a specific moment of the day-night cycle. Nudibranchs, snails and sea-stars are also predators of deep-sea gorgonian corals [105]. Their presence activity may progressively increase over the seabed at daytime in our study site, affecting *Paragorgia*’s behavior.

In this scenario, the applied multivariate statistic ANN modelling approach demonstrated a non-linear dependence of *Paragorgia* polyp extrusion in relation to some combination of oceanographic variables. Bloating was correlated with chlorophyll concentrations over consecutive months as a proxy for seasonality (see Figure 13), which cannot be confirmed here for the lack of longer time series of biological data. Sherwood et al. [106] identified that that a main food source for CWC is fresh phytodetritus. Fresh chlorophyll inputs can be transported to deep-sea canyon areas by tidal pulses on a 24-h basis and high concentrations can be detected below photic and despotic areas [107]. Furthermore, during the study period, polyp extrusion activity (i.e., the bloated status likely associated to filtering) increased from March to June being associated with an incremental increase in chlorophyll concentration. This would agree with previous observations on shallow water octocorals, where polyp extrusion rates seasonally fluctuated depending on overall food inputs [108,109,110].

## 5. Conclusions

Our results reaffirm that unexplored underwater environments can be analyzed with the help of traditional computer vision approaches combined with deep learning approaches. In doing so, we established a model for the development of highly integrated pipelines for the extraction of biological information from times series images and consequent online treatment of multidisciplinary information at cabled observatory infrastructures. The computer vision approaches for the improvement and enhancement of underwater image quality plays an important role that, when combined with techniques aimed at the classification of relevant subjects, can produce relevant operational advances in the ecological monitoring capability of cabled observatories.

## Figures and Tables

**Table 1:** Oceanographic variables and sensors used at the LoVe monitoring site for statistic comparison with *P. arborea* polyp extrusion/activity rhythms.

Sensor	Model
Pressure and temperature	Aanderaa 4117D
Salinity	Aanderaa 4319A
Turbidity	Aanderaa 4112A
Chlorophyll	WetLabs BFL2W

**Table 2:** Precision Accuracy (PA) and User Accuracy (UA) of each of the 4 state categories.

States	PA	UA
Bloated	82.85%	80.97%
Non-Bloated	61.57%	61.57%
Semi-Bloated	65.04%	65.04%
Background	87.36%	87.36%

**Table 3:** Time series parameters as absolute maximum and minimum as well as significant (*p* > 0.05) periods (both in total minutes (Min) and hours (h)) for all behavioral and oceanographic variables measured at the LoVe observatory in 2018. The Midline Estimated Statistic of a Rhythm (MESOR) is also reported as a result from waveform analysis for all the time series.

				Period	
	Max	Min	MESOR	Min	h
Bloated	99.97	4.43	60.56	1435	23.9
Non-Bloated	87.07	0.00	21.42	1435	23.9
Semi-Bloated	67.86	0.00	18.02	1435	23.9
Temperature	7.31	5.61	6.52	Not Done	
Salinity	35.00	34.23	34.74	Not Done	
Chlorophyll	26.24	0.32	7.05	Not Done	
Turbidity	124.28	5.56	6.58	Not Done	
Depth	251.15	248.88	250.10	Not Done	

**Table 4:** Characteristics and principal results of the Multi-Layer Feed forward artificial neural Network (MLFN) model (training and internal test) in predicting the coral activity status (i.e., bloated vs. non-bloated) for the training and the test sets.

**Training (70%)**	
Number of cases	1150
Number of hidden layers	1
Number of nodes	9
Training time	0:7:02
Number of trials	500
% bad predictions	0.26 (3)
**Testing (30%)**	
Number of cases	503
% bad predictions (N)	31 (156)

**Table 5:** Confusion matrix of the test set of the MLFN model used in predicting the classification bloated versus non-bloated activity statuses. Targets are reported on rows. The correctly classified samples are reported on the main diagonal of the matrix.

	Bloated	Non-Bloated	Total
Bloated	223	70	293
Non-Bloated	86	124	210
